# Computational Analysis of Immunohistochemistry Enables Quantitative Assessment of SARS-CoV-2 Nucleocapsid Immunolabeling in Experimentally Infected Mice

**DOI:** 10.3390/v18101142

**Published:** 2026-10-14

**Authors:** Nedim Kozarac, Marius Botos, Laura Burgener, Simone de Brot, Francisco Brito, Inês Berenguer Veiga, Adriano Taddeo, Christelle Devisme, Stefano Bagatella, Nadine Ebert, Güliz Tuba Barut, Étori Aguiar Moreira, Nathan George François Leborgne, Melanie Brügger, Volker Thiel, Charaf Benarafa, Llorenç Grau-Roma

**Affiliations:** 1 Institute of Virology and Immunology, Sensemattstrasse 293, 3147 Mittelhäusern, Switzerland; nedim.kozarac@ivi.admin.ch (N.K.); marius.botos@unibe.ch (M.B.); francisco.brito@ivi.admin.ch (F.B.); inesmbveiga@gmail.com (I.B.V.); devisme.christelle@gmail.com (C.D.); nadine.ebert@ivi.admin.ch (N.E.); gueliz.barut@ivi.admin.ch (G.T.B.); etori.aguiarmoreira@ivi.admin.ch (É.A.M.); nathan.leborgne@york.ac.uk (N.G.F.L.); melanie.bruegger@ivi.admin.ch (M.B.); volker.thiel@unibe.ch (V.T.); charaf.benarafa@ivi.admin.ch (C.B.); 2 Department of Infectious Diseases and Pathobiology, Vetsuisse Faculty, University of Bern, Länggassstrasse 122, 3012 Bern, Switzerland; laura.burgener@unibe.ch (L.B.); simone.debrot@unibe.ch (S.d.B.);; 3 Graduate School for Cellular and Biomedical Sciences, University of Bern, Länggassstrasse 122, 3012 Bern, Switzerland; 4 COMPATH, Institute of Animal Pathology, Vetsuisse Faculty, University of Bern, Länggassstrasse 122, 3012 Bern, Switzerland; 5 Multidisciplinary Center for Infectious Diseases (MCID), University of Bern, Länggassstrasse 122, 3012 Bern, Switzerland

**Keywords:** digital histopathology, quantitative pathology, SARS-CoV-2, immunohistochemistry

## Abstract

Histopathology and immunohistochemistry (IHC) are central to COVID-19 tissue evaluation. However, conventional manual scoring is limited by its semiquantitative nature and potential observer variability. In this retrospective study of experimentally infected mice, we implemented a semi-automated digital pathology workflow using commercially available software to quantitatively assess SARS-CoV-2 nucleocapsid (N) immunolabeling in lung (*n* = 135) and brain (*n* = 67) tissues. The performance of digital quantification was evaluated against conventional manual scoring, and its association with virological and pathological parameters was assessed across different stages of disease progression. Digital IHC quantification demonstrated strong agreement with manual scoring in both lung [R = 0.94, *p* < 0.0001, concordance correlation coefficient (CCC) = 0.95] and brain [R = 0.98, *p* < 0.0001; CCC = 0.977], demonstrating concordance between the two approaches. In addition, digital SARS-CoV-2 N immunolabeling quantification showed significant positive correlations with viral RNA levels, infectious viral titers, and histopathological scores, supporting its association with independent measures of SARS-CoV-2 infection and lesions. Although computational image analysis requires additional infrastructure, technical expertise, and increased analysis time, it provides an objective continuous quantitative measure of SARS-CoV-2 N immunolabeling that complements conventional manual assessment and may reduce observer-dependent variability.

## 1. Introduction

Severe Acute Respiratory Syndrome Coronavirus 2 (SARS-CoV-2) is a recently emerged RNA virus belonging to the family Coronaviridae [1]. Due to its high transmissibility and replication efficiency, the virus rapidly spread worldwide, leading to a global pandemic in 2020 [1,2]. Clinical manifestations of coronavirus diasease-19 (COVID-19) are highly variable [3] and commonly include fever, cough, sore throat, and temporary loss of smell and/or taste, among other symptoms that may resemble those of influenza. In severe cases, COVID-19 can progress to pneumonia, acute respiratory distress syndrome, and multi-organ failure [4]. In that regard, animal models have been central to advancing our understanding of COVID-19, enabling the study of host–pathogen interactions, immune responses, and the evaluation of vaccines and therapeutics in an in vivo context [5]. Because susceptibility of wild-type mice to SARS-CoV-2 infection varies among viral variants, and murine angiotensin-converting enzyme 2 (ACE2) generally supports viral entry less efficiently than human ACE2, several complementary mouse models have been used to study coronavirus infection, each capturing distinct aspects of the disease. The Keratin 18–human ACE2 (K18-hACE2) transgenic model is characterized by severe, often lethal disease with high viral replication in the upper respiratory tract (URT) and lungs. While it is highly useful for vaccine and antiviral efficacy studies due to its robust phenotype, it is limited by frequent and extensive brain infection [6,7,8], which is disproportionate to the extent of central nervous system (CNS) infection generally observed in human COVID-19 [9,10]. In contrast, hACE2 knock-in (hACE2-ki) mice express human ACE2 under endogenous control, with lower ACE2 expression in the lungs than K18-hACE2 mice and limited CNS involvement, resulting in a milder disease phenotype. Despite their milder phenotype, hACE2-ki mice support viral replication in the respiratory tract and can be used to evaluate antivirals and to compare emerging variants with their parental strains in vivo [11]. Mouse-adapted SARS-CoV-2 strains, such as MA10, provide an alternative approach that enables infection of wild-type mice and induces severe respiratory disease with high viral loads, supporting antiviral screening and mechanistic studies [12,13]. These models therefore provide complementary experimental systems, with their different disease phenotypes, tissue tropism and genetic backgrounds allowing investigation of SARS-CoV-2 pathogenesis, antiviral efficacy, and host determinants of disease [14].

Immunohistochemistry (IHC) has been widely used to detect SARS-CoV-2 proteins in tissue sections and to map viral distribution in infected organs, particularly in postmortem material from both human and animal origin. In many SARS-CoV-2 studies, IHC positivity is still assessed using manual semiquantitative scoring systems, which remain a widely used reference approach due to their familiarity, practicality, and integration into routine pathology workflows [15,16,17]. While pathology literature regards manual semiquantitative scoring as the traditional gold standard, it also recognizes its inherent limitations, including subjectivity, restricted dynamic range, and variability in reproducibility compared with digital approaches [18,19,20]. Aiming to overcome these limitations, there is currently a clear shift toward digital image analysis and automated quantification. Several studies have already used digital pathology platforms to quantify SARS-CoV-2 antigen as well as other stains [18,19,21], showing that objective computational assessment is feasible in this setting. Nevertheless, a systematic comparison of digital IHC quantification with manual semiquantitative assessment has not yet been established in mouse models of SARS-CoV-2 infection.

In this study, we aimed to evaluate the suitability of a digital image analysis framework for quantitative assessment of SARS-CoV-2 nucleocapsid (N) immunostaining in lung and brain samples by comparing it with traditional semiquantitative scoring methods. As a secondary objective, we evaluated the association of digital quantification of SARS-CoV-2 N immunohistochemical staining with histopathological and virological parameters to assess its utility for measuring disease severity in experimentally infected mice.

## 2. Materials and Methods

### 2.1. Sample Selection

All brain (*n* = 67) and lung (*n* = 135) tissues analyzed were from archived materials previously collected from mice across 27 independent experiments grouped into 12 experimental cohorts, some already published [7,12,22,23,24] or in preparation (Supplementary File S1). A total of 186 mice of both sexes were included in the analysis. All mice were inoculated intranasally with SARS-CoV-2 doses ranging from 10^2^ to 10^6^ TCID_50_ per mouse. Intranasal infections were administered at a volume of 20 µL per nostril. Samples were collected from 1 to 15 days post-infection (dpi) and grouped into three phases of infection based on previous works [8,25,26]: early infection (1–4 dpi; lung, *n* = 45 [33%]; brain, *n* = 6 [9%]), peak of infection (5–7 dpi; lung, *n* = 86 [64%], brain, *n* = 55 [82%]) and resolution phase (8–15 dpi; lung, *n* = 3 [2%], brain, *n* = 6 [9%]). Since most samples were collected at the peak of infection (5–7 dpi), all time points were grouped together for the analyses.

### 2.2. SARS-CoV-2 Mouse Models

Mouse experiments were conducted in compliance with the Swiss Animal Welfare legislation and were approved by the Commission for Animal Experimentation of the Cantonal Veterinary Office of Bern under license numbers BE43/2020 and BE68/2023. Mice were housed and bred at the specific-pathogen-free mouse facility at the Institute of Virology and Immunology, where they were maintained in individually ventilated cages (Blue line, Tecniplast, Buguggiate, Italy), with 12 h/12 h light/dark cycle, 22  ±  1 °C ambient temperature and 50  ±  5% humidity, autoclaved food and acidified water. At least 7 days before infection, mice were placed in individually HEPA-filtered cages (IsoCage N, Tecniplast).

Samples were collected from inbred mice (C57BL/6J, BALB/c, 129/Sv), outbred (SWISS) and genetically modified models: B6J-Tg(K18-hACE2)2Prlmn (K18-hACE2), CB6F1-Tg(K18-hACE2)2Prlmn, B6.Cg-Ace2tm1(ACE2)Dwnt/J, K18-hACE2.Casp1tm1Sesh/Casp11129mt, Gsdmd.Gsdmeem1Cben.

Mice were infected with SARS-CoV-2 stocks generated by TAR cloning as previously described [27] or, when indicated, derived from clinical isolates. The strains included the ancestral Wuhan strain (GenBank ID: MN908947.3), the SARS-CoV-2 SD614G ancestral strain with the D614G substitution in spike [24], and SARS-CoV-2 variants Alpha (BioProject ID: PRJNA784099), Alpha isolate (received from the Robert-Koch-Institut, Berlin, Germany), Beta (BioProject ID: PRJNA784099), Delta (BioProject ID: PRJNA868423), Omicron BA.1 (BioProject ID: PRJNA868423), BA.5 (BioProject ID: PRJNA827255), and MA10 (GenBank ID: MT952602.1).

### 2.3. Grouping of Results

This study focused on evaluating the feasibility and utility of digital IHC quantification as a tool in SARS-CoV-2 research compared to manual semiquantitative IHC assessment. Additionally, to assess the biological relevance of the quantitative approach, digitally quantified IHC positivity was compared with virological and histopathological scores obtained from the same organs, including viral RNA levels (qRT-PCR), infectious viral titers (VT) and histopathological scores. Viral strain differences were not considered in this analysis to focus on broader correlational trends across infection dynamics. RT-qPCR values and infectious VT were log10-transformed for analysis and reported as viral E gene copies/mL and VT (TCID_50_/mL or PFU/mL), respectively. Organ samples with undetermined cycle threshold (Ct) values or fewer than 1500 RNA copies/mL were considered RT-qPCR negative based on a standard curve [28]. Samples below the limit of detection (<10^1^ TCID_50_/mL) in the cytopathic effect (CPE) assay were also considered negative, as were those showing no CPE in any replicate wells [29]. Missing data and non-detected values were handled using a complete-case approach. Specimens with missing values were excluded from the corresponding correlation analysis prior to statistical testing. Supplementary File S1 detailed information on the available samples, the tests performed and their results and selected experimental information for each mouse included in the study.

### 2.4. Histopathological Analysis

Lung (left lung: *n* = 125; entire lung *n* = 10) and brain (mid-sagittal brain half; *n* = 56; entire brain: *n* = 11) were fixed in 10% neutral buffered formalin, embedded in paraffin, cut at 4 μm of thickness and stained with hematoxylin and eosin (H&E). H&E-stained lung and brain slides were examined and scored blindly by an ECVP board-certified veterinary pathologist (LGR). Lungs were assessed using a comprehensive scoring scheme described previously [11], while brains were scored from 0 to 3 as follows: 0 = no lesions; 1 = focal or multifocal perivascular infiltrate by up to 1 layer of leukocytes, small areas of gliosis or mild neutrophilic infiltrates; 2 = few multifocal areas of gliosis or inflammation, with perivascular cuffs of 1 to 3 layers of leukocytes; 3 = moderate to abundant multifocal areas of inflammation and gliosis, with prominent perivascular cuffs (Supplementary Figure S1).

### 2.5. Immunohistochemistry (IHC)

For IHC, 3 μm formalin-fixed, paraffin-embedded lung and brain sections were mounted on positively charged slides, dried for 30 min at 60 °C and subsequently dewaxed, pretreated and stained on an Immunostainer Leica Bond RX (Leica Biosystems, Mount Waverley, Australia). IHC was performed using anti-SARS-CoV-2 nucleocapsid (N) rabbit antibodies, either polyclonal (Rockland, 200-401-A50, Limerick, PA, USA) or monoclonal (Cell Signaling, #26369, clone HL344, Danvers, MA, USA). The IHC procedure using the polyclonal (Rockland) antibody was performed as previously described [6]. After dewaxing (Bond Dewax solution; Leica Biosystems), the procedure with the monoclonal (Cell Signaling) antibody included an antigen retrieval step in citrate buffer (Bond Epitope Retrieval 1; pH 6) for 30 min at 100 °C. The slides were then incubated with the monoclonal antibody diluted at 1:5000 for 30 min at room temperature. All further steps were performed using reagents from the Bond Polymer Refine Detection Kit (Leica Biosystems, Mount Waverley, Australia) as follows: endogenous peroxidase was blocked for 5 min, then a mouse anti-rabbit secondary antibody was applied (15 min), followed by a peroxidase-labeled polymer (15 min). Finally, slides were developed in 3,3′-diaminobenzidine (DAB)/H_2_O_2_ (10 min), counterstained with hematoxylin, and mounted.

### 2.6. Digital IHC Analysis

**Figure 1:**
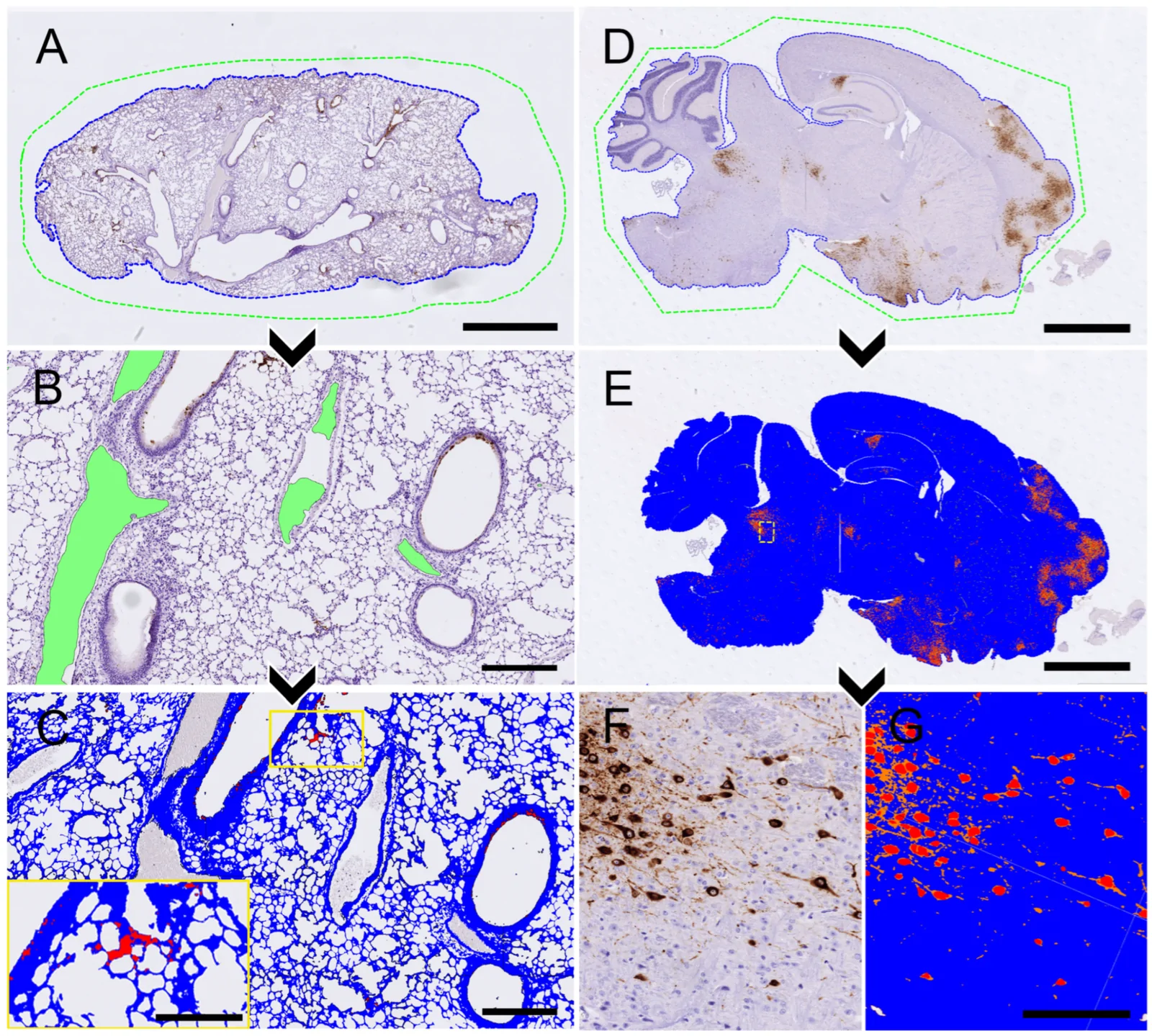
Image analysis workflow with Visiopharm software. Lung (**left**) and brain (**right**) with immunohistochemical staining against SARS-CoV-2 nucleocapsid (N) from whole slide section images. (**A**,**D**) Tissue detection. The area containing lung (**A**) or brain (**D**) tissue is first manually marked (green discontinuous line), and subsequently, the tissue within it is automatically detected by the tissue detection Application Protocol Package (APP) (blue discontinuous line). (**B**) Erythrocyte exclusion in the lung. Large aggregates of erythrocytes typically present within the blood vessels are detected to be excluded from the analysis (marked with filled green areas) using a deep learning (DL) APP. (**C**) SARS-CoV-2 N immunolabeling detection in the lung. A threshold pixel-based classification APP is used to detect immunohistochemically positive (red labels) from negative lung parenchyma (blue labels), with exclusion of empty spaces. Inset: high magnification of the area marked with a yellow rectangle. (**D**–**G**) SARS-CoV-2 N immunolabeling detection in the brain. A combined DL-threshold classification APP is used to detect the positively stained neuronal soma (red labels), the remaining immunohistochemical staining (orange labels) and negative brain parenchyma (blue). Images (**F**,**G**) correspond to a higher magnification of the area marked with a yellow rectangle in image (**E**), without and with labels, respectively. Scale bars: (**A**,**D**,**E**): 2000 µm, (**B**,**C**): 350 µm; (**G**,**C**): 150 µm.

Visiopharm software (Version 2024.07, Hørsholm, Denmark) was used to perform quantitative digital histological analysis. The IHC glass slides were digitized with a NanoZoomer S360 digital slide scanner (Hamamatsu Photonics K.K., Hamamatsu City, Japan) for whole slide image (WSI) analysis. WSIs were produced at a resolution of 40×, corresponding to 0.23 µm/pixel. The generated files were in the ‘.ndpi’ format. Each lung or brain section was defined as a ‘region of interest’ (ROI), which was fully assessed using different Application Protocol Packages (APPs) as described below and illustrated in Figure 1. All slides were analyzed using the same APPs within each tissue.

(1) Tissue detection. A pixel-based decision forest classifier at 1× input magnification was used to detect the available lung or brain tissue on the slide and to outline the generated tissue label as ‘region of interest’ (ROI) for subsequent analysis. The classifier was trained based on a subset of slides (slide training set) of 20 WSI (*n* = 10 lung, *n* = 10 brain). For each training slide, a single ROI of 1–3 mm^2^ was defined and manually annotated for two classes: lung tissue vs. background.
(2) Erythrocyte exclusion in the lung. A pixel-wise deep learning (DL) (U-Net architecture) classifier at 10× magnification and based on 140,000 training iterations was used to detect and exclude tissue areas with large aggregates of intravascular erythrocytes. This step prevented these blood cell clusters from being counted as negative lung parenchyma, which would otherwise result in an underestimation of the proportional IHC staining. The classifier was trained based on a slide training set of 13 WSI. For each training slide, a single ROI of 1–3 mm^2^ was defined and manually annotated for two classes: erythrocyte aggregates vs. background.
(3) SARS-CoV-2 N immunolabeling detection in the lung. A pixel-based threshold classifier at 20× input magnification was implemented using a combination of two color features: FastRed_DAB-DAB and Green (where 0 represents maximum color intensity and 255 represents white). Pixels with a FastRed_DAB-DAB value < 95 were classified as IHC positive. Within the remaining tissue, pixels were defined as IHC-negative lung parenchyma if they exhibited a FastRed_DAB-DAB value *≥* 95 and a Green value < 220. Combining these features allowed us to exclude empty alveolar and vascular spaces, which were characterized by Green feature values of ≥220. Thresholds were visually established through inspection by two pathologists (LGR and SdB) and subsequently applied to all slides. SARS-CoV-2 IHC-positive tissue area was quantified both as an absolute area (mm^2^) and as a percentage of the total assessed lung tissue area.
(4) SARS-CoV-2 N immunolabeling detection in the brain. A hybrid classification approach at 20× input magnification was implemented to detect both somal and extracellular immunolabeling. First, a pixel-based U-Net DL model was trained for 157,000 iterations using 10 IHC slides (3–5 manually annotated ROIs of approximately 0.01 mm^2^ per slide) to calculate the pixel-wise probability of neuronal somata against the surrounding neuropil. This probability feature was then integrated into a pixel-based decision workflow alongside color features (FastRed_DAB-DAB and Green; intensity scale: 0 = maximum intensity, 255 = white). Pixels were labeled as one of the following label classes based on the given definitions: IHC-Positive Neuronal Somata: Pixels with a DL soma probability *≥* 0.95. Extracellular IHC-Positive Tissue: Pixels with a DL soma probability < 0.95 and a FastRed_DAB-DAB value < 90. Extracellular IHC-Negative Tissue: Pixels with a DL soma probability < 0.95, a FastRed_DAB-DAB value *≥* 90, and a Green value < 228. Pixels exhibiting Green values *≥* 228 were classified as empty background spaces and excluded. All color thresholds were visually established by two pathologists (LGR and SdB). Although the pipeline allows independent quantification of neuronal somata, SARS-CoV-2 N positivity was reported and analyzed as the total percentage of positive tissue area (combining somal and extracellular positivity) relative to the total assessed brain tissue area per section, consistent with the approach used for the lung.
(5) In all analytic steps, manual revision and corrections of the generated ROIs and labels were performed in each slide before generating the results. Manual corrections were minor, took up to 5 min per slide, and consisted mostly of excluding tissue artefacts. Staining and tissue processing artefacts causing false-positive labeling were excluded manually, marking them as ‘clear ROIs’.

### 2.7. Manual IHC Analysis

Manual semiquantitative scores were performed by two authors (LGR and LB). The criteria used were based on previously reported manual scoring systems in both the lung [30] and brain [31] with minor modifications. The scores were defined as follows: 0 = negative; 1 = focal to few multifocal positivity present in single cells or few small aggregates; 2 = low to moderate multifocal positivity present in small cell aggregates; 3 = moderate multifocal to coalescing positivity; and 4 = abundant multifocal to coalescing or diffuse positivity.

### 2.8. RT-qPCR and Virus Titration Assays

Samples were collected and processed as described before [7,11]. Briefly, samples were taken from the superior right lobe (RT-qPCR) and middle, inferior, and post-caval lobes (VT) of the lung and from the frontal half of the right brain hemisphere (RT-qPCR and VT). Total RNA extraction was performed with the NucleoMag VET kit (Macherey- Nagel, Düren, Germany). Viral copies were determined by RT-qPCR for the viral E gene transcript as described previously [32]. Interpolated values for viral RNA copies in 5 µL of the reaction mix were used. These values were then extrapolated to RNA copies/mL. Infectious VT of SARS-CoV-2 were determined by plaque assay in Vero E6 cells (Vero C1008, ATCC) (*n* = 41, 20 lung and 21 brain) or TCID_50_ assay in Vero/TMPRSS2 (NIBSC Research Reagent Depository, UK) cells (*n* = 161, 115 lung and 46 brain), as previously described [7,11]. Virus titers obtained by plaque assay and reported as PFU/mL were subsequently converted to TCID_50_/mL as previously reported [33] to allow comparison with titers obtained by TCID_50_ assay. Conversion was performed on the log_10_ scale using the relationship 1 PFU ≈ 0.69 TCID_50_, corresponding to a subtraction of 0.161 from the log_10_ PFU/mL value.

### 2.9. Statistical Analysis and Data Visualization

Statistical analyses were performed using R (version 4.4.0) in RStudio (version 2026.1.1.403) [34]. R session information, including packages used, is provided in Supplementary File S2. Manual IHC scoring agreement analyses between the two raters (LGR and LB) were performed on pairwise complete observations for both lung and brain tissues. Three complementary metrics were used: quadratic weighted Cohen’s kappa (k) using irr package [35], Lin’s concordance correlation coefficient (CCC) using DescTools [36], and Spearman’s rank correlation coefficient (R) using cor.test and Hmisc::rcorr [37]. Quadratic weighting was selected because the scoring scale (0–4) is ordinal and differences between categories represent increasing biological severity. The quadratic weighting scheme penalizes larger disagreements more strongly than minor ones. CCC was used to quantify overall concordance by jointly assessing accuracy (closeness to the identity line) and precision (strength of linear association), providing a single measure of reproducibility that is robust for bounded ordinal numeric scales. Spearman’s rank correlation coefficient (R) was included to evaluate monotonic association between raters without assuming linearity.

Correlation analyses were performed using Spearman’s two-sided correlation coefficient (R) to assess monotonic relationships between variables. The significance of correlations was determined using a two-sided hypothesis test with a Student’s t-approximation for the distribution of the test statistic, as implemented in the ‘rcorr’ function from the Hmisc package in R. This approach accounts for ties in the data. Statistical significance was defined as follows: *p* ≥ 0.05: not significant (ns), *p* < 0.05: *, *p* < 0.01: **, *p* < 0.001: ***.

CCC analysis was used to quantify the agreement between manual and digital scoring across experimental groups, which jointly evaluates precision and accuracy by measuring the deviation of paired observations from the 45° line of perfect concordance [32]. CCC values were calculated for each group and returned with their respective 95% confidence intervals. CCC requires both measurements to be expressed on comparable scales. Manual scores were recorded on a discrete 0–4 ordinal scale, whereas values from the digital analysis were continuous percentages. To harmonize these scales, we used randomized regression trees using the rpart package to learn data-driven cut points [38]. This method relies on an iterative partitioning process that minimizes the variance within splits of the continuous data. To prevent overfitting and ensure an unbiased estimation of the cutpoints, we incorporated a 5-fold cross-validation procedure using the caret package [39]. This rotatory approach separates the dataset into 5 parts (4/5 training and 1/5 for validation), and it was used to train an rtree model 5 times, always leaving a part of the dataset out for validation and never contributing to its own cutoff. Based on these optimized splits, the continuous values were assigned to discrete regions. This resulted in digital quantitative values that were converted into discrete categories corresponding to the manual scoring system.

Agreement and interchangeability between manual scores and digital results were evaluated using Bland–Altman analysis [40]. Digital quantitative values were harmonized into the same 0–4 ordinal scale as the manual score using the rtree-based binning procedure described above, making both methods directly comparable. For each sample, differences were calculated between manual scores and the digital (binned) ordinal values and their mean. The bias was determined as the average of these differences, representing the systematic offset of the methods. The limits of agreement (LoA) were calculated as bias ± 1.96 × SD (differences), indicating the range within which 95% of individual differences are expected to fall. This provided a direct measure of whether the two scoring methods could be considered interchangeable at the individual-sample level, where small LoA values may support interchangeability.

To evaluate the consistency of the relationship between manual scoring and digital quantification results while accounting for experimental heterogeneity, independent linear mixed-effects models were implemented for lung and brain tissues using the lme4 [41] and lmerTest [42] packages in R. For each model, the manual score was specified as a fixed effect, whereas the unique data source (defined as the individual publication or unpublished experimental cohort; individual experiments belonging to the same overall study were grouped within the same cohort) was treated as a random intercept factor. These cohorts shared key experimental characteristics and provided a more appropriate grouping for capturing study-level heterogeneity, while reducing the number of small group sizes that would result from treating each individual experiment as a separate cohort (Supplementary File S1). This mixed-effects framework explicitly models cohort-level clustering, thereby accounting for shared baseline technical and biological variation across different study batches. Denominator degrees of freedom and *p*-values for the fixed effects were calculated using Satterthwaite’s approximation. To further validate the consistency of these patterns within individual cohorts, repeated-measures correlation analyses (rmcorr package, [43]) were performed separately for each tissue. This secondary framework utilized the data source as the independent grouping variable to assess the common, parallel within-study linear association between manual scoring and digital image quantification.

## 3. Results

### 3.1. Excellent Agreement Between Observers in Manual Scoring

Both observers presented an excellent agreement across the full dataset. Quadratic weighted k was 0.972/0.991 for lung and brain, respectively, indicating an almost perfect agreement on the categorical 0–4 scale. CCC was 0.972/0.991, demonstrating a very high concordance with very small systematic bias between raters. Spearman’s correlation was 0.974/0.994 with a *p*-value < 0.0001, reflecting a strong relationship between the two scoring sets. Further statistical analyses were performed by using the scores of the ECVP board-certified pathologist.

### 3.2. Digital Quantification of SARS-CoV-2 N Shows Strong Concordance with Manual Quantification in Both Lung and Brain Tissues

**Figure 2:**
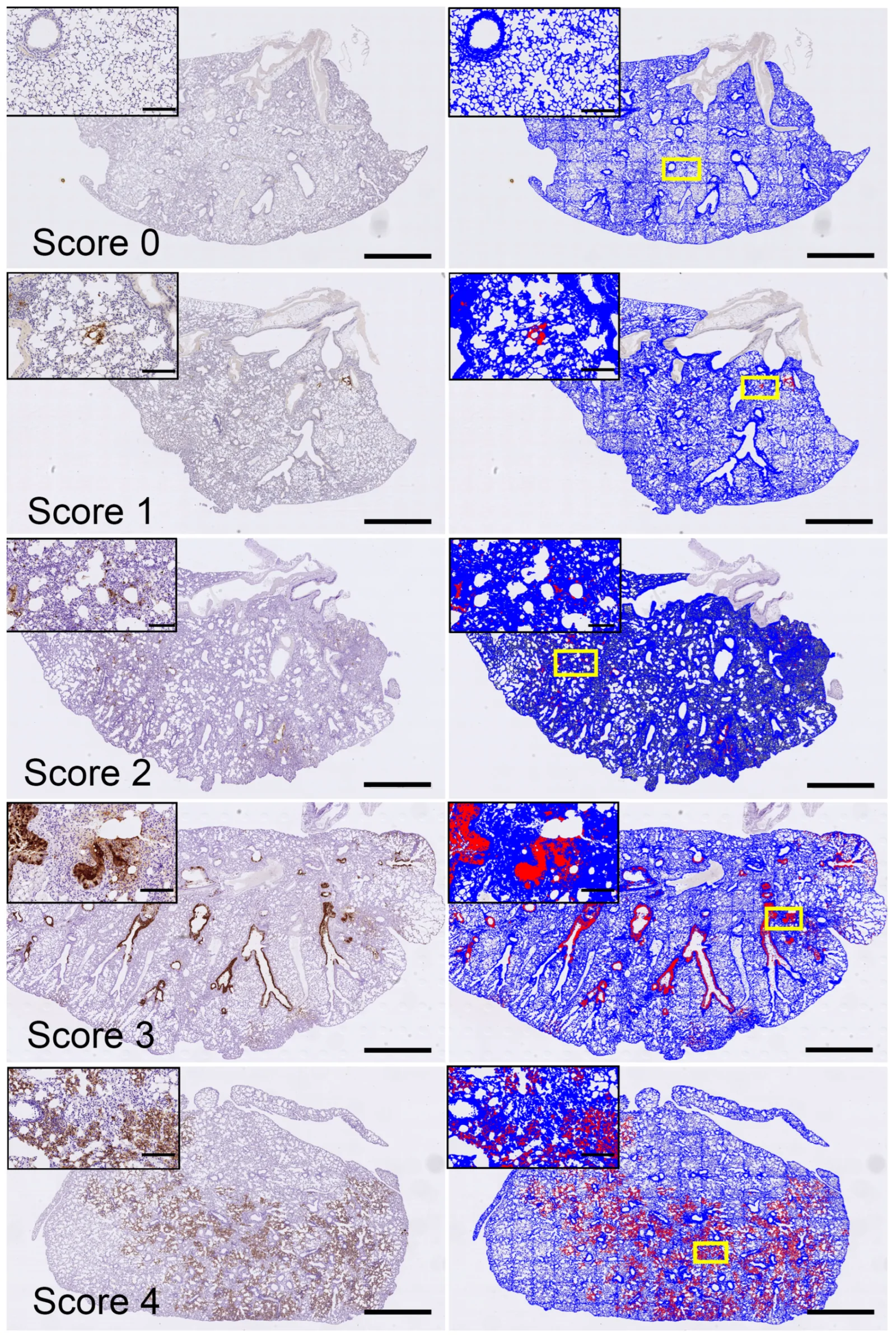
Representative images of the lung scoring scheme. Images on the left depict the immunohistochemistry for SARS-CoV-2 nucleocapsid from lungs with manual scores ranging from 0 to 4 (**top**–**bottom**), while figures on the right are the corresponding same images, including the digital labeling of the positive (red) and negative (blue) lung parenchyma. Scale bar: 1500 µm. Insets of each image correspond to higher-magnification views of the area marked with a yellow rectangle in the corresponding digitally labeled image. Scale bar in the insets: 150 µm.

**Figure 3:**
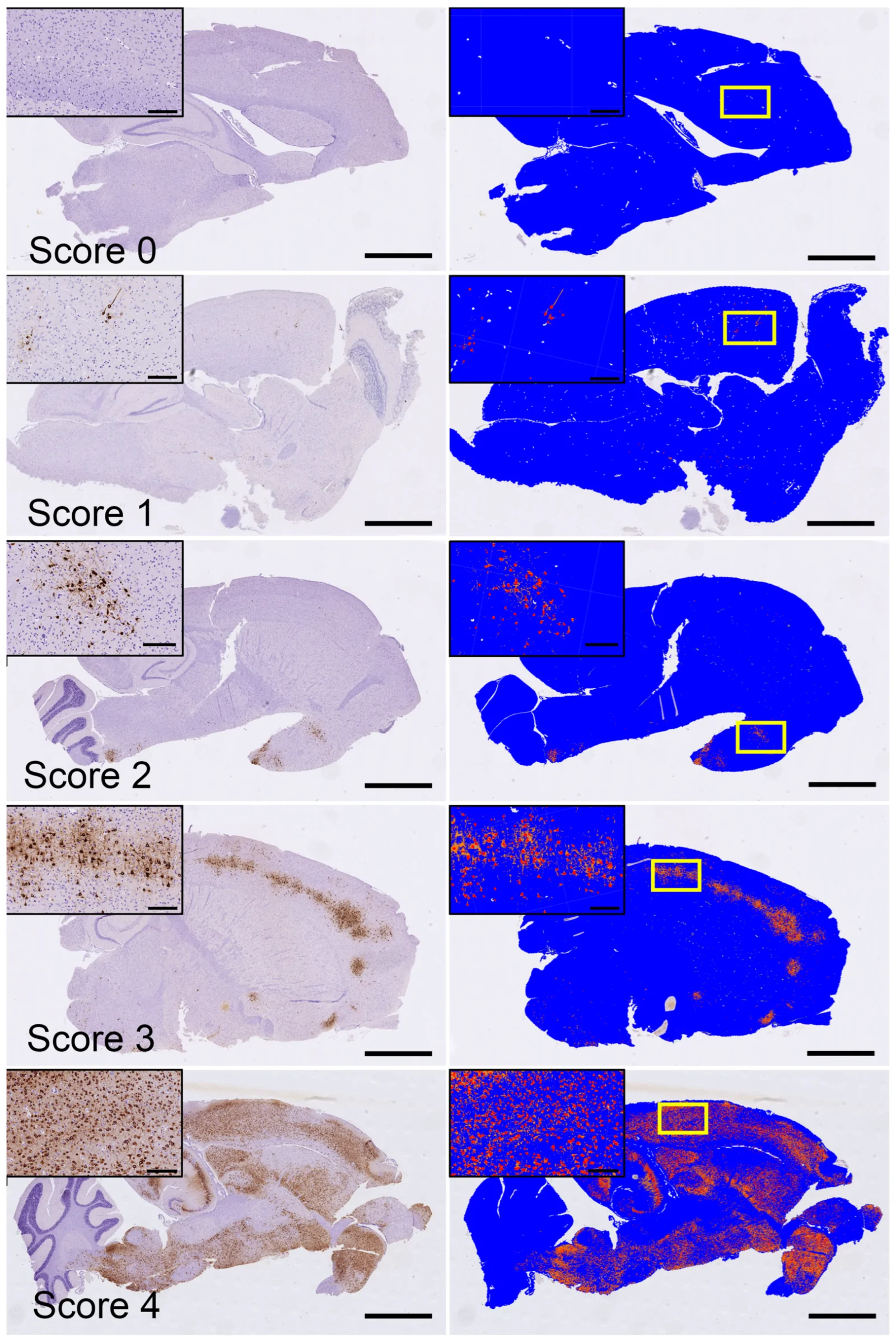
Representative images of the brain scoring scheme. Images on the left depict the immunohistochemistry for SARS-CoV-2 nucleocapsid from brains with manual scores ranging from 0 to 4 (**top**–**bottom**), while figures on the right are the corresponding same images, including the digital labeling of the positive neuronal soma (red), the remaining positivity (orange) and the negative (blue) brain parenchyma. Scale bar: 1500 µm. Insets of each image correspond to higher-magnification views of the area marked with a yellow rectangle in the corresponding digitally labeled image. Scale bar in the insets: 150 µm.

Figure 2 and Figure 3 present representative examples comparing manual scoring with the corresponding automated detection of the SARS-CoV-2 N immunoreactivity in lung and brain, respectively. When assessed separately, the two antibodies produced comparable staining patterns (Supplementary Figure S2), with similar correlations between digital quantification and manual scores in lung tissue for the monoclonal and polyclonal antibodies (R = 0.96, *p* < 0.001 and R = 0.91, *p* < 0.001, respectively). Only a minority of brain sections were stained with the polyclonal antibody, and all were negative, precluding a meaningful comparison between antibodies in brain tissue. For the subsequent analyses, results obtained with both antibodies were considered together for each tissue.

**Figure 4:**
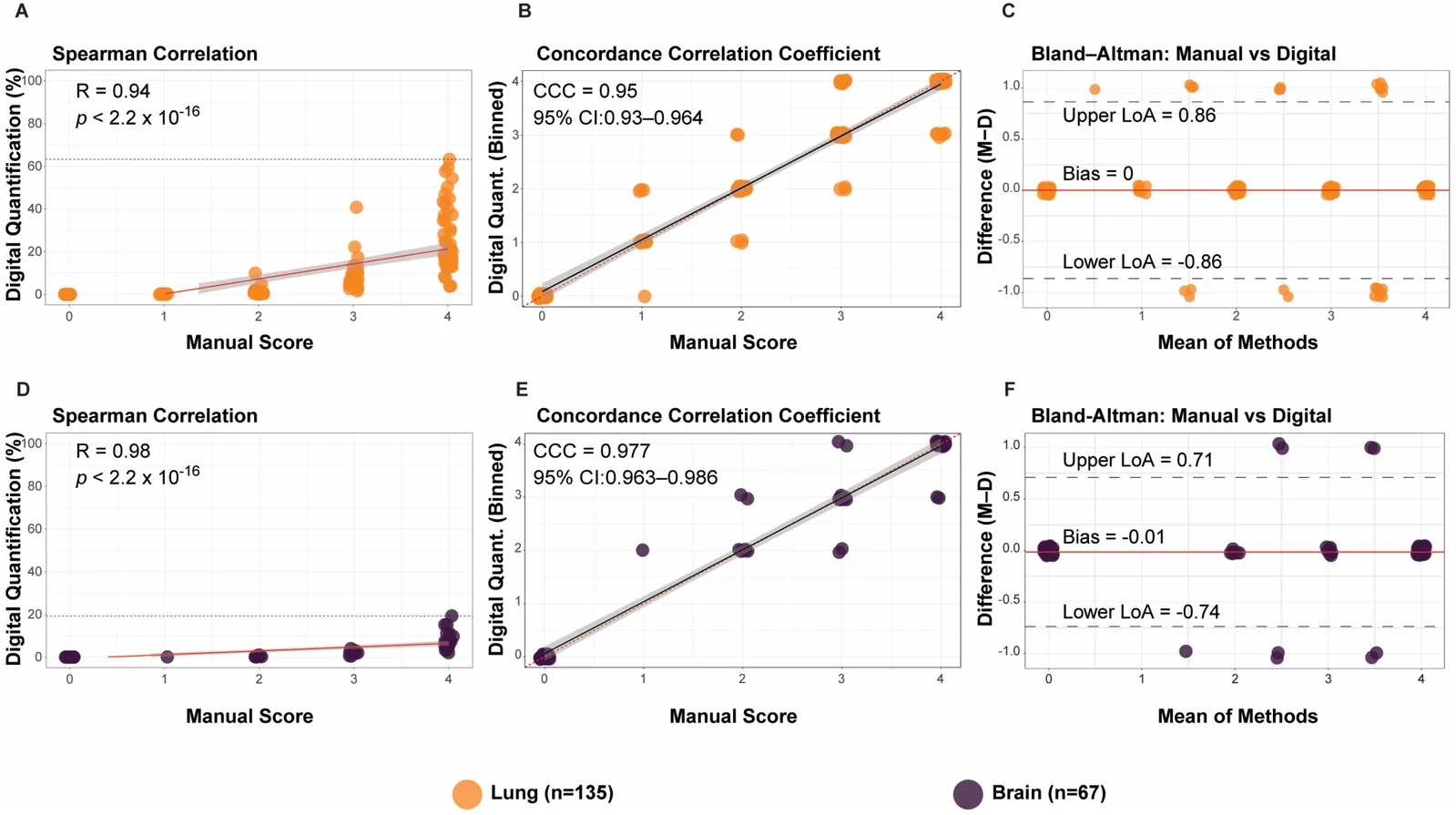
Digital IHC quantification shows strong agreement with manual scoring in lungs and brain. (**A**,**B**) Correlation (Spearman’s R) and concordance correlation coefficient (CCC) analysis between digital SARS-CoV-2 immunohistochemical stain quantification results and manual IHC scores in lung tissue (*n* = 135). (**C**) Bland–Altman analysis of agreement in lung tissue. Limits of agreement (LoA) represent the range within which 95% of the differences between the two methods fall. (**D**,**E**) Correlation and CCC analysis between digital SARS-CoV-2 immunohistochemical stain quantification results and manual IHC scores in brain tissue (*n* = 67). (**F**) Bland–Altman analysis of agreement in brain tissue.

In the lung, digital immunohistochemical stain quantification results showed strong positive correlations with manual IHC scores by Spearman analysis (R = 0.94, *p* < 0.001) (Figure 4A). Digital quantification closely mirrored manual scoring, demonstrating excellent consistency in ranking SARS-CoV-2 N immunopositivity across samples. CCC analysis demonstrated a high agreement between digital values (binned) and manual scores (CCC = 0.95, 95% CI: 0.93–0.964) (Figure 4B), indicating near-perfect agreement, with measurements closely matching both in trend and absolute values. Bland–Altman analysis revealed a negligible mean bias (0), indicating the absence of a systematic difference between manual and digital (binned) scoring. However, the limits of agreement were relatively wide (–0.86 to +0.86), suggesting that individual measurements may vary by up to approximately 20% (~0.8 scoring units from a 0–4 scale) (Figure 4C).

Similar to the lung, digital immunohistochemical stain quantification results in the brain showed an almost perfect correlation with manual scoring (R = 0.98, *p* < 0.001) (Figure 4D). CCC analysis in the brain samples further confirmed near-complete agreement between the two methods (CCC = 0.977, 95% CI: 0.963–0.986) (Figure 4E). Here, the Bland–Altman analysis demonstrated a minimal bias (−0.01) and slightly narrower limits of agreement of approximately 20% (–0.74 to +0.71), indicating good concordance between manual and digital scoring (Figure 4F). Furthermore, to evaluate the association between manual scores and the digital values while accounting for the experimental cohort to which each mouse belonged, we analyzed brain (six experimental cohorts) and lung (10 experimental cohorts) samples (Supplementary File S1) separately using two complementary approaches: repeated-measures correlation [lung tissue (R = 0.74, *p* = 8.48 × 10^−12^) and brain tissue (R = 0.66, *p* = 4.23 × 10^−17^)], and mixed-effects models [lung *p* < 2 × 10^−16^ and brain *p* < 2.8 × 10^−12^]. Both approaches demonstrated that the association between manual and digital values remained strong and statistically significant after accounting for the source of measurement.

### 3.3. Digital IHC Scoring Shows Good Correlation with Histological Lesions and Virological Data

To get insights into the biological relevance of the digitally quantified percentage of SARS-CoV-2 N-positive area, we next examined its association with RT-qPCR measures of viral load and histopathology. Correlation analyses were performed using both original continuous digital results and the corresponding binned values to assess whether binning altered the observed relationships. The same correlations were also performed with the manual scores to allow comparison between both methods.

**Figure 5:**
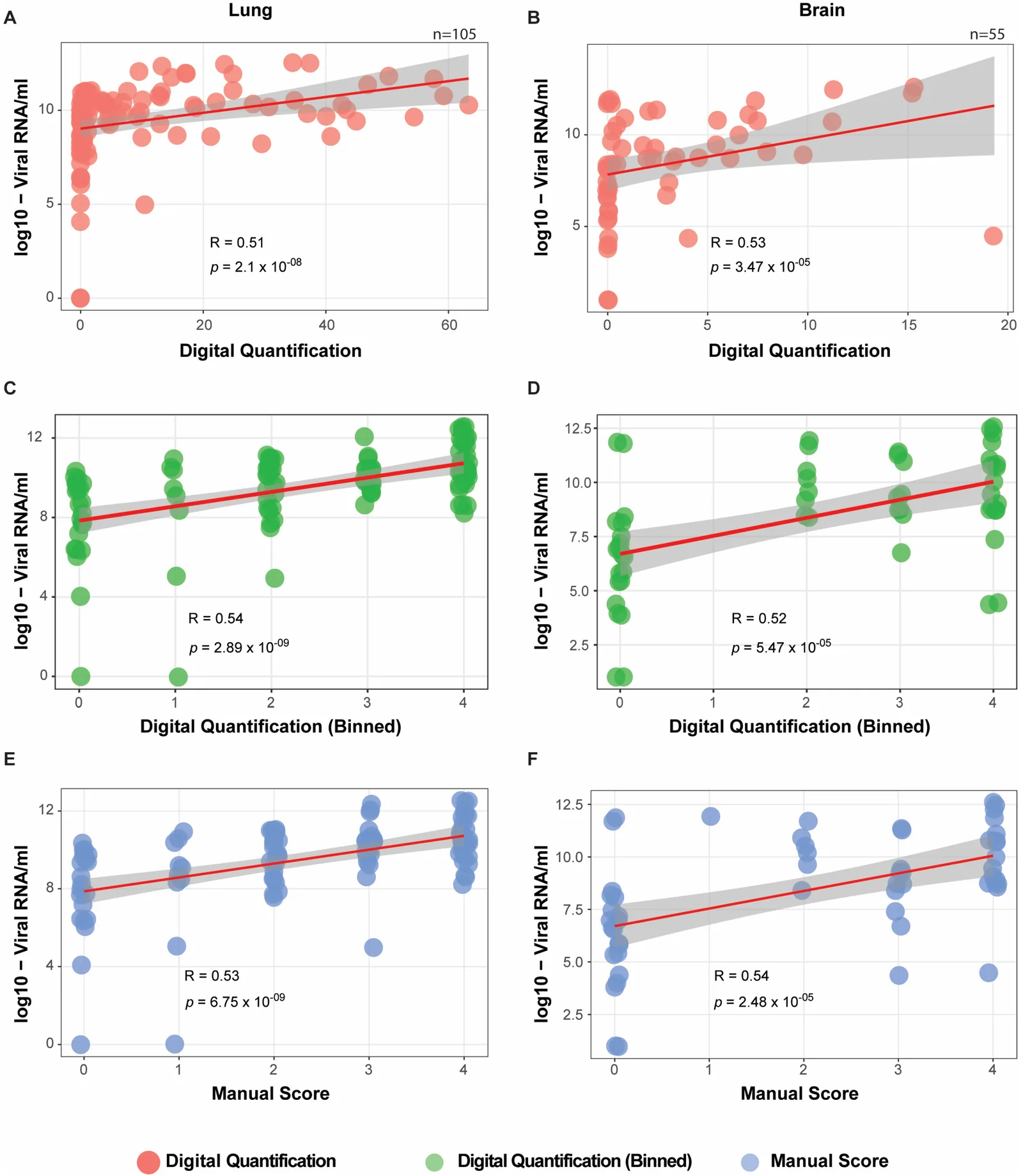
Digital values of SARS-CoV-2 nucleocapsid immunolabeling correlate with viral RNA in lung and brain. (**A**–**F**) Correlations between viral RNA and digital IHC values (**A**,**B**), binned digital IHC values (**C**,**D**), or manual IHC scores (**E**,**F**) are shown for the lungs (**A**,**C**,**E**) and brains (**B**,**D**,**F**). Viral RNA was quantified by RT–qPCR targeting the SARS-CoV-2 E gene and is presented as log10 viral RNA copies/mL. Spearman correlation coefficients (R) and corresponding *p* values are indicated in each panel. Correlations were assessed using two-sided Spearman rank correlation, with *p* values calculated using the asymptotic t-approximation because of tied observations.

Analysis of viral RNA levels revealed similar, moderate positive correlations with digital immunohistochemical stain quantification results in both lung (R = 0.51, *p* < 0.001) and brain (R = 0.53, *p* < 0.001). Correlation analysis using binned digital IHC values and manual scores showed a very similar pattern (Figure 5).

**Figure 6:**
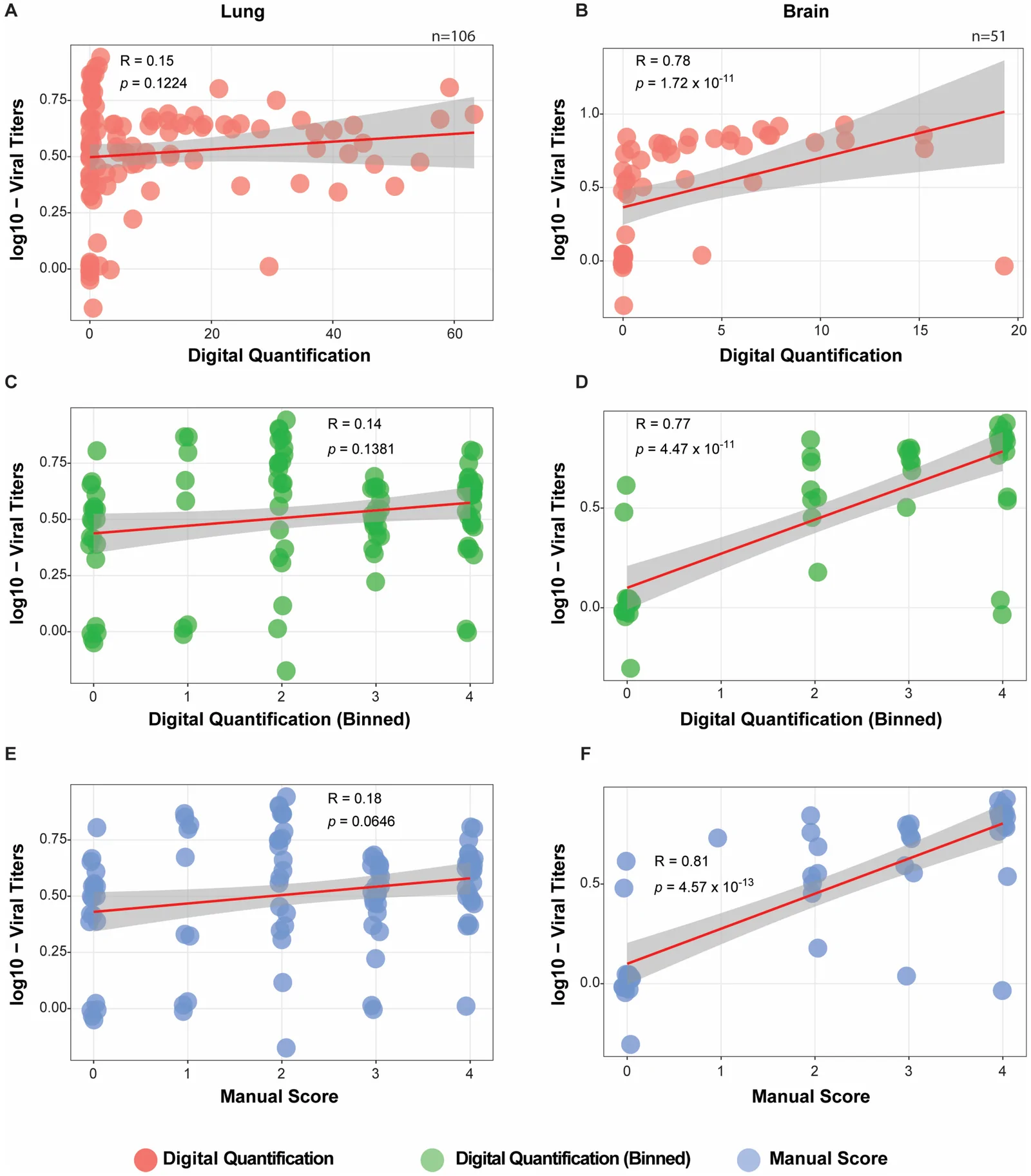
Digital values of SARS-CoV-2 nucleocapsid immunolabeling correlate with live virus titers in brain but not in lung of SARS-CoV-2 infected mice. (**A**–**F**) Correlations between live virus titers and digital IHC values (**A**,**B**), binned digital IHC values (**C**,**D**), or manual IHC scores (**E**,**F**) are shown for the lungs (**A**,**C**,**E**) and brain (**B**,**D**,**F**). Infectious viral titers were determined by TCID_50_ or plaque assay. To harmonize VT across both assays, PFU/mL values from plaque assays were converted to TCID_50_/mL using the relationship 1 PFU ≈ 0.69 TCID_50_ [33]. Spearman correlation coefficients (R) and corresponding *p* values are indicated in each panel. Correlations were assessed using two-sided Spearman rank correlation, with *p* values calculated using the asymptotic t-approximation because of tied observations.

Analysis of VT demonstrated a significant positive correlation with digital IHC results in the brain (R = 0.78, *p* < 0.001), while such correlation was not seen in the lung (R = 0.15, *p* = 0.12). These results were consistent with the binned digital IHC results and manual scores (Figure 6).

**Figure 7:**
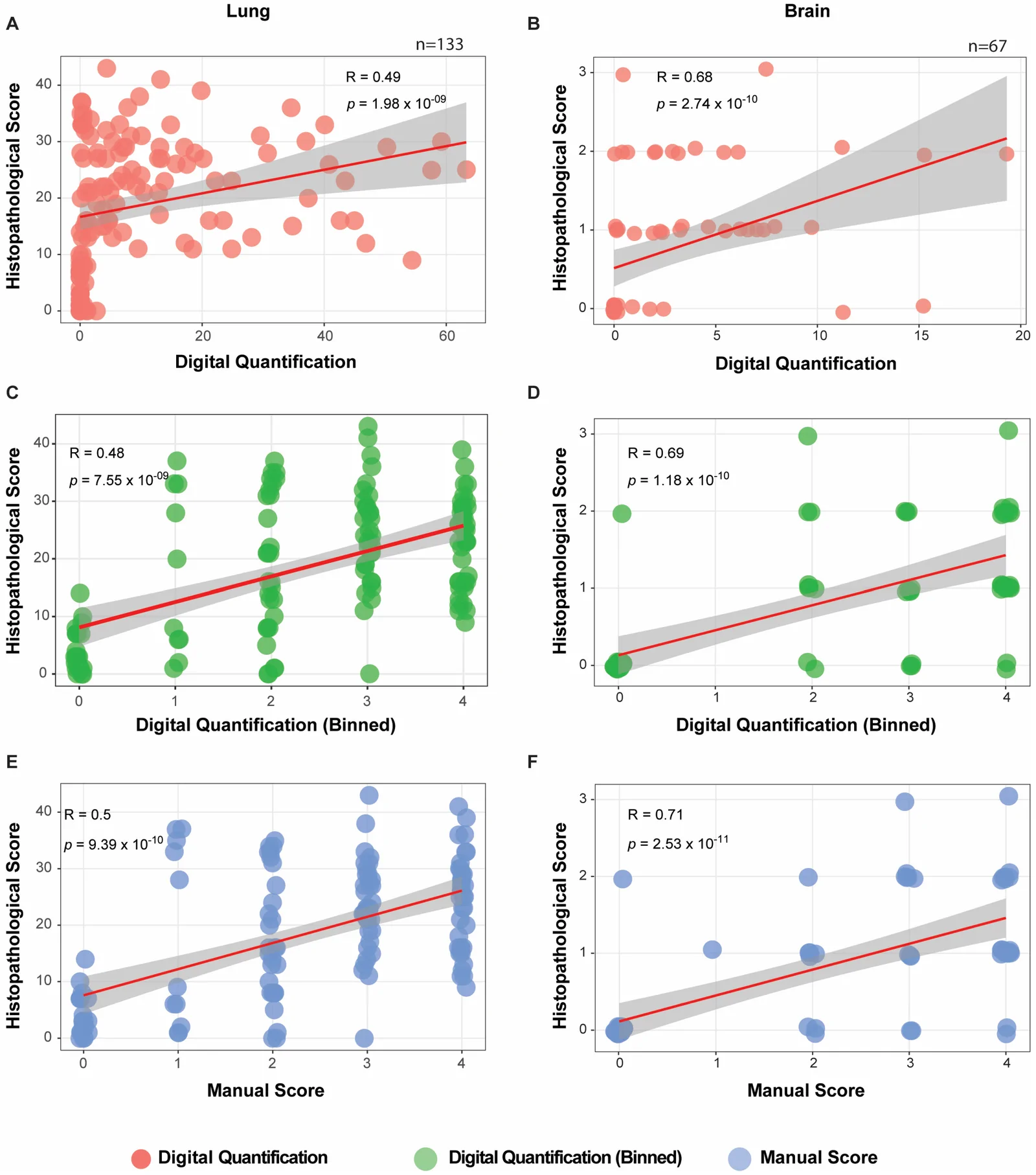
Digital values of SARS-CoV-2 nucleocapsid immunolabeling correlate with histopathological scores in lung and brains. (**A**–**F**). Correlations between histopathological scores and digital IHC results (**A**,**B**), binned digital IHC values (**C**,**D**), or manual IHC scores (**E**,**F**) are shown for the lungs (**A**,**C**,**E**) and brain (**B**,**D**,**F**). Spearman correlation coefficients (R) and corresponding *p* values are indicated in each panel. Correlations were assessed using two-sided Spearman rank correlation, with *p* values calculated using the asymptotic t-approximation because of tied observations.

Finally, digital IHC results showed a moderate positive correlation with the histopathological scores in both lung (R = 0.49, *p* < 0.001) and brain (R = 0.68, *p* < 0.001). Correlation analysis using binned digital IHC values and manual scores showed again a very similar pattern (Figure 7).

## 4. Discussion

In this study, we retrospectively evaluated a digital image analysis approach for quantifying SARS-CoV-2 N immunostaining in lung and brain tissues from experimentally infected naïve mice. While manual scoring remains widely used, it is inherently semi-quantitative and subject to inter- and intra-observer variability [44,45]. In contrast, digital IHC analysis provides continuous quantitative assessment of immunohistochemically positive areas and may reduce observer-dependent variability [44,45]. Notably, the pipeline implemented here quantifies IHC positivity within the organ parenchyma, excluding empty spaces (i.e., pulmonary airspaces, cerebrospinal fluid spaces, and vascular lumina), thereby mitigating potential variation related to disease severity (e.g., emphysema and atelectasis), processing factors (inflated vs. non-inflated lung), and processing artefacts. The very strong correlations observed between digital immunostaining quantification and manual IHC scoring indicate strong agreement between digital and manual IHC assessments, consistent with findings from other studies in infectious diseases as well as in other fields such as oncology, where digital pathology is widely established [46,47]. This strong agreement indicates that the extent of immunohistochemical staining is an important component of the manual assessment performed by experienced observers. Digital quantification measures the extent of staining as continuous data, providing greater quantitative resolution than categorical assessments and reducing the subjectivity associated with assigning ordinal scores. Importantly, however, the high concordance between the two methods does not imply that they are interchangeable, as they provide continuous and ordinal measurements, respectively.

By performing Bland–Altman analysis, we observed minimal bias between manual and digital assessments for both brain and lung tissues, indicating good agreement. However, the limits of agreement remain relatively wide, suggesting that the two scoring approaches may yield method-dependent differences under certain conditions. This, together with the semiquantitative and quantitative nature of manual and digital methods, respectively, indicates that both methods are not fully interchangeable. This is further illustrated by the overlap in digitally quantified immunostaining positivity among samples assigned to different manual scores, particularly scores 2–4. Such overlap is expected in an ordinal, semiquantitative system, in which broad categories do not correspond to strict quantitative cut-offs and may also reflect subjective assessment and potential inter- and intra-observer variability [18]. Altogether, our results indicate that the two approaches provide complementary rather than interchangeable information.

An additional advantage of digital quantification was that it provided a quantitative measure of the extent of SARS-CoV-2 N-positive tissue involvement, which is not readily captured by ordinal manual scores. In our dataset, the proportion of SARS-CoV-2 N-positive lung parenchyma reached approximately 60%, compared with approximately 20% in the brain. This difference is consistent with the lung being the predominant site of SARS-CoV-2 infection following intranasal exposure, while brain infection can also occur in K18-hACE2 mice, particularly during severe disease [8,48].

The good correlation between SARS-CoV-2 N immunohistochemical positivity and most of the assessed independent measures of viral burden and histological damage indicates an association between digital immunolabeling quantification and the assessed histological lesions and virological parameters. Most animals included in this study were euthanized during the peak of infection (5–7 dpi), and the findings therefore primarily reflect this stage, which is characterized by high levels of viral replication and extensive tissue involvement [48,49]. Importantly, RT-qPCR, viral titration, and IHC quantify related but distinct aspects of infection: viral RNA, infectious virus, and tissue immunopositivity for viral N protein, respectively. Consequently, individual measurements may not show a one-to-one correspondence, as illustrated by the lack of correlation between lung VT and viral antigen abundance. This may reflect both the different biological parameters measured and sampling mismatch, as viral titration and IHC were performed on different lung lobes, together with the spatially heterogeneous and multifocal distribution of SARS-CoV-2 within the lungs following intranasal inoculation [48,50]. The observed correlations may differ during earlier stages of infection, when viral RNA or infectious virus may already be detectable in tissues before the onset of histopathological lesions [48]. Likewise, during the resolution phase, viral replication declines substantially, and infectious viruses are markedly reduced or absent, whereas residual or defective viral genetic material may still be detected [51].

Digital IHC assessment provides quantitative, continuous measurement of viral antigen burden. When combined with spatial analysis, this approach could further enable assessment of the spatial relationship between antigen distribution and tissue lesions, thereby linking viral presence to histological changes. Despite these strengths, several limitations should be considered. The retrospective design of this study entailed variability in several parameters, including tissue processing and immunohistochemical staining conditions such as the antibodies used and potential batch-to-batch variability, which may influence digital quantification [45,52]. Nevertheless, the comparable staining patterns and similar correlations between digital and manual assessments observed for the two antibodies in lung tissue support the consistency of the digital analysis across the staining protocols used. Similarly, the use of different virus titration assays and cell lines represents an additional source of methodological variability. Although PFU/mL and TCID_50_/mL values were harmonized by conversion, differences in the underlying titration methods and cell lines may still contribute to variability in viral titer measurements and, consequently, to the observed correlations. Nevertheless, in more standardized and homogeneous study settings, the consistency of digital quantification is expected to be even higher than that observed here. Notably, the digital quantification was semi-automated since it still required supervision, meaning review of each slide and manual correction when necessary.

Digital quantification was performed using a single digital pathology platform and image-analysis workflow, as comparison across platforms was beyond the scope of this study. This ensured consistent application across all samples. Although previous studies have demonstrated good agreement between digital pathology platforms, quantitative results may still be influenced by differences in algorithms, image-processing parameters, and analytical workflows [53,54]. The present findings therefore establish the performance of the specific platform and workflow evaluated here, while further studies could assess its reproducibility across independent platforms and workflows.

An important consideration for the implementation of digital pathology is the additional investment in infrastructure, technical expertise, and time compared with conventional manual assessment. Digital image analysis requires access to a slide scanner, ideally capable of generating high-resolution WSIs, as well as adequately powered computer hardware, dedicated image analysis software and the corresponding technical expertise. In addition, it must be acknowledged that digital workflow is associated with increased processing time per slide, including scanning, image preprocessing, and computational analysis, which in this case exceed the time required for manual scoring by an experienced observer. The manual analysis was performed in seconds for clearly positive cases (scores > 1) and up to approximately two minutes for weakly positive cases and negative (score of 1). Consequently, although digital analysis offers advantages in terms of continuous quantitative data generation and reduced observer dependence, these benefits should be balanced against the additional infrastructure, expertise, and operational requirements associated with its implementation.

Future studies could increase cohort sizes, include additional time points, and explore advanced image analysis approaches, including segmentation of distinct tissue compartments, to assess SARS-CoV-2 immunopositivity within specific anatomical regions and provide more detailed spatial information. For instance, this framework could be applied not only to intranasal infection models but to nebulized infection models, to quantify SARS-CoV-2 immunostaining across different infection settings. The approach may also be extended to other viral pathogens, provided the appropriate immunohistochemical assays and image-analysis parameters are established and validated. In the present study, digital IHC provided continuous quantitative measurements of SARS-CoV-2 N staining that complemented conventional semiquantitative pathological assessment. Taken together, these findings support digital IHC as a useful quantitative approach for assessing tissue viral immunopositivity, with potential for further integration with additional spatial and tissue-specific analyses.

## Data Availability

The original contributions presented in this study are included in the article. Further inquiries can be directed to the corresponding author.

## Supplementary Materials

The following supporting information can be downloaded at: https://www.mdpi.com/article/10.3390/v18101142/s1, File S1: Available results and detailed experimental information for each mouse included in the study; File S2: R session information used for statistical analyses, including R and RStudio versions, system information, and versions of the packages loaded during the analyses; Figure S1: Representative images of the brain histopathological scoring system; Figure S2: Comparison of immunohistochemical staining with two different antibodies against the SARS-CoV-2 nucleocapsid protein.

## Abbreviations

The following abbreviations are used in this manuscript:

ACE2	Angiotensin-converting enzyme 2
APP	Application Protocol Package
CCC	Concordance correlation coefficient
CNS	Central nervous system
COVID-19	Coronavirus diasease-19
DAB	3,3′-diaminobenzidine
CPE	Cytopathic effect
DL	Deep learning
dpi	Days post-infection
ECVP	European college of veterinary pathologists
FFPE	Formalin-fixed, paraffin-embedded
H&E	Hematoxylin and eosin
IHC	Immunohistochemistry
K18-hACE2	Keratin 18–human angiotensin-converting enzyme 2
LoA	Limits of agreement
MA10	Mouse-adapted 10
N	Nucleocapsid
ROI	Region of interest
SARS-CoV-2	Severe acute respiratory syndrome coronavirus 2
URT	Upper respiratory tract
VT	Viral titers
WSI	Whole slide image

## References

[1] Lai C.C., Shih T.P., Ko W.C., Tang H.J., Hsueh P.R. (2020). Severe acute respiratory syndrome coronavirus 2 (SARS-CoV-2) and coronavirus disease-2019 (COVID-19): The epidemic and the challenges. Int. J. Antimicrob. Agents.

[2] Li R., Pei S., Chen B., Song Y., Zhang T., Yang W., Shaman J. (2020). Substantial undocumented infection facilitates the rapid dissemination of novel coronavirus (SARS-CoV-2). Science.

[3] Larsen J.R., Martin M.R., Martin J.D., Kuhn P., Hicks J.B. (2020). Modeling the Onset of Symptoms of COVID-19. Front. Public Health.

[4] Singhal T. (2020). A Review of Coronavirus Disease-2019 (COVID-19). Indian J. Pediatr..

[5] Fan C., Wu Y., Rui X., Yang Y., Ling C., Liu S., Liu S., Wang Y. (2022). Animal models for COVID-19: Advances, gaps and perspectives. Signal Transduct. Target. Ther..

[6] Schon J., Barut G.T., Trueb B.S., Halwe N.J., Berenguer Veiga I., Kratzel A., Ulrich L., Kelly J.N., Brugger M., Wylezich C. (2024). A safe, effective and adaptable live-attenuated SARS-CoV-2 vaccine to reduce disease and transmission using one-to-stop genome modifications. Nat. Microbiol..

[7] Taddeo A., Veiga I.B., Devisme C., Boss R., Plattet P., Weigang S., Kochs G., Thiel V., Benarafa C., Zimmer G. (2022). Optimized intramuscular immunization with VSV-vectored spike protein triggers a superior immune response to SARS-CoV-2. npj Vaccines.

[8] Vidal E., Lopez-Figueroa C., Rodon J., Perez M., Brustolin M., Cantero G., Guallar V., Izquierdo-Useros N., Carrillo J., Blanco J. (2022). Chronological brain lesions after SARS-CoV-2 infection in hACE2-transgenic mice. Vet. Pathol..

[9] Khan M., Clijsters M., Choi S., Backaert W., Claerhout M., Couvreur F., Van Breda L., Bourgeois F., Speleman K., Klein S. (2022). Anatomical barriers against SARS-CoV-2 neuroinvasion at vulnerable interfaces visualized in deceased COVID-19 patients. Neuron.

[10] Stein S.R., Ramelli S.C., Grazioli A., Chung J.-Y., Singh M., Yinda C.K., Winkler C.W., Sun J., Dickey J.M., Ylaya K. (2022). SARS-CoV-2 infection and persistence in the human body and brain at autopsy. Nature.

[11] Ulrich L., Halwe N.J., Taddeo A., Ebert N., Schon J., Devisme C., Trueb B.S., Hoffmann B., Wider M., Fan X. (2022). Enhanced fitness of SARS-CoV-2 variant of concern Alpha but not Beta. Nature.

[12] Grin P.M., Baid K., de Jesus H.C.R., Kozarac N., Bell P.A., Jiang S.Z., Kappelhoff R., Butler G.S., Leborgne N.G.F., Pan C. (2024). SARS-CoV-2 3CL(pro) (main protease) regulates caspase activation of gasdermin-D/E pores leading to secretion and extracellular activity of 3CL(pro). Cell Rep..

[13] Leborgne N.G., Devisme C., Kozarac N., Berenguer Veiga I., Ebert N., Godel A., Grau-Roma L., Scherer M., Plattet P., Thiel V. (2024). Neutrophil proteases are protective against SARS-CoV-2 by degrading the spike protein and dampening virus-mediated inflammation. JCI Insight.

[14] Knight A.C., Montgomery S.A., Fletcher C.A., Baxter V.K. (2021). Mouse Models for the Study of SARS-CoV-2 Infection. Comp. Med..

[15] Krasemann S., Dittmayer C., von Stillfried S., Meinhardt J., Heinrich F., Hartmann K., Pfefferle S., Thies E., von Manitius R., Aschman T.A.D. (2022). Assessing and improving the validity of COVID-19 autopsy studies—A multicentre approach to establish essential standards for immunohistochemical and ultrastructural analyses. EBioMedicine.

[16] Sun Y., Ge L., Udhane S.S., Langenheim J.F., Rau M.J., Patton M.D., Gallan A.J., Felix J.C., Rui H. (2021). Sensitive and Specific Immunohistochemistry Protocol for Nucleocapsid Protein from All Common SARS-CoV-2 Virus Strains in Formalin-Fixed, Paraffin Embedded Tissues. Methods Protoc..

[17] Usai C., Pailler-Garcia L., Lorca-Oro C., Fernandez-Bastit L., Roca N., Brustolin M., Rodon J., Perez M., Cantero G., Carrillo J. (2022). Agreement and differential use of laboratory methods for the detection and quantification of SARS-CoV-2 in experimentally infected animals. Front. Microbiol..

[18] Meyerholz D.K., Beck A.P. (2018). Principles and approaches for reproducible scoring of tissue stains in research. Lab. Investig..

[19] Wu H., He P., Ren Y., Xiao S., Wang W., Liu Z., Li H., Wang Z., Zhang D., Cai J. (2022). Postmortem high-dimensional immune profiling of severe COVID-19 patients reveals distinct patterns of immunosuppression and immunoactivation. Nat. Commun..

[20] Zehntner S.P., Chakravarty M.M., Bolovan R.J., Chan C., Bedell B.J. (2008). Synergistic tissue counterstaining and image segmentation techniques for accurate, quantitative immunohistochemistry. J. Histochem. Cytochem..

[21] Piedra-Mora C., Robinson S.R., Tostanoski L.H., Dayao D.A.E., Chandrashekar A., Bauer K., Wrijil L., Ducat S., Hayes T., Yu J. (2022). Reduced SARS-CoV-2 disease outcomes in Syrian hamsters receiving immune sera: Quantitative image analysis in pathologic assessments. Vet. Pathol..

[22] Barut G.T., Halwe N.J., Taddeo A., Kelly J.N., Schon J., Ebert N., Ulrich L., Devisme C., Steiner S., Trueb B.S. (2022). The spike gene is a major determinant for the SARS-CoV-2 Omicron-BA.1 phenotype. Nat. Commun..

[23] Túrós D., Gladiseva L., Botos M., He C., Barut G.T., Veiga I.B., Ebert N., Bonnin A., Chanfon A., Grau-Roma L. (2025). Deep learning-based 3D spatial transcriptomics with X-Pression. bioRxiv.

[24] Zhou B., Thao T.T.N., Hoffmann D., Taddeo A., Ebert N., Labroussaa F., Pohlmann A., King J., Steiner S., Kelly J.N. (2021). SARS-CoV-2 spike D614G change enhances replication and transmission. Nature.

[25] An H.W., Kim H.A., Ha T.H., Seok S.H., Lee Y.J., Lee N.Y., Yoon G.M., Choi Y.K., Lee H.Y., Yoo W.G. (2026). Deep learning-assisted, pathogenesis-informed lung histopathology scoring in preclinical mouse models of SARS-CoV-2 and influenza A infection. Front. Immunol..

[26] Carossino M., Kenney D., O’Connell A.K., Montanaro P., Tseng A.E., Gertje H.P., Grosz K.A., Ericsson M., Huber B.R., Kurnick S.A. (2022). Fatal Neurodissemination and SARS-CoV-2 Tropism in K18-hACE2 Mice Is Only Partially Dependent on hACE2 Expression. Viruses.

[27] Thi Nhu Thao T., Labroussaa F., Ebert N., V’Kovski P., Stalder H., Portmann J., Kelly J., Steiner S., Holwerda M., Kratzel A. (2020). Rapid reconstruction of SARS-CoV-2 using a synthetic genomics platform. Nature.

[28] Corman V.M., Landt O., Kaiser M., Molenkamp R., Meijer A., Chu D.K., Bleicker T., Brunink S., Schneider J., Schmidt M.L. (2020). Detection of 2019 novel coronavirus (2019-nCoV) by real-time RT-PCR. Eurosurveillance.

[29] Ramakrishnan M.A. (2016). Determination of 50% endpoint titer using a simple formula. World J. Virol..

[30] de Campos-Mata L., Trinite B., Modrego A., Tejedor Vaquero S., Pradenas E., Pons-Grifols A., Rodrigo Melero N., Carlero D., Marfil S., Santiago C. (2024). A monoclonal antibody targeting a large surface of the receptor binding motif shows pan-neutralizing SARS-CoV-2 activity. Nat. Commun..

[31] Sanchez-Morales L., Porras N., Garcia-Seco T., Perez-Sancho M., Cruz F., Chinchilla B., Barroso-Arevalo S., Diaz-Frutos M., Buendia A., Moreno I. (2024). Neuropathological lesions in intravenous BCG-stimulated K18-hACE2 mice challenged with SARS-CoV-2. Vet. Res..

[32] Lin L.I. (1989). A concordance correlation coefficient to evaluate reproducibility. Biometrics.

[33] ATCC ATCC Virology Culture Guide. https://www.atcc.org/en/resources/culture-guides/virology-culture-guide.

[34] Posit Team (2026). RStudio: Integrated Development Environment for R, 2026.1.1.403.

[35] Gamer M., Lemon J., Fellows I., Singh P. (2026). irr: Various Coefficients of Interrater Reliability and Agreement, 0.85.

[36] Signorell A. (2025). DescTools: Tools for Descriptive Statistics, 0.99.60.

[37] Harrell F.E. (2026). Hmisc: Harrell Miscellaneous, 5.2-5.

[38] Speybroeck N. (2012). Classification and regression trees. Int. J. Public Health.

[39] Kuhn M. (2008). Building Predictive Models in R Using the caret Package. J. Stat. Softw..

[40] Bland J.M., Altman D.G. (1986). Statistical methods for assessing agreement between two methods of clinical measurement. Lancet.

[41] Bates D., Mächler M., Bolker B., Walker S. (2015). Fitting Linear Mixed-Effects Models Usinglme4. J. Stat. Softw..

[42] Kuznetsova A., Brockhoff P.B., Christensen R.H.B. (2017). lmerTest Package: Tests in Linear Mixed Effects Models. J. Stat. Softw..

[43] Bakdash J.Z., Marusich L.R. (2017). Repeated Measures Correlation. Front. Psychol..

[44] Bernardo V., Lourenco S.Q., Cruz R., Monteiro-Leal L.H., Silva L.E., Camisasca D.R., Farina M., Lins U. (2009). Reproducibility of immunostaining quantification and description of a new digital image processing procedure for quantitative evaluation of immunohistochemistry in pathology. Microsc. Microanal..

[45] Chlipala E.A., Bendzinski C.M., Dorner C., Sartan R., Copeland K., Pearce R., Doherty F., Bolon B. (2020). An Image Analysis Solution For Quantification and Determination of Immunohistochemistry Staining Reproducibility. Appl. Immunohistochem. Mol. Morphol..

[46] Rizzardi A.E., Johnson A.T., Vogel R.I., Pambuccian S.E., Henriksen J., Skubitz A.P., Metzger G.J., Schmechel S.C. (2012). Quantitative comparison of immunohistochemical staining measured by digital image analysis versus pathologist visual scoring. Diagn. Pathol..

[47] Sood A., Sui Y., McDonough E., Santamaria-Pang A., Al-Kofahi Y., Pang Z., Jahrling P.B., Kuhn J.H., Ginty F. (2020). Comparison of Multiplexed Immunofluorescence Imaging to Chromogenic Immunohistochemistry of Skin Biomarkers in Response to Monkeypox Virus Infection. Viruses.

[48] Winkler E.S., Bailey A.L., Kafai N.M., Nair S., McCune B.T., Yu J., Fox J.M., Chen R.E., Earnest J.T., Keeler S.P. (2020). SARS-CoV-2 infection of human ACE2-transgenic mice causes severe lung inflammation and impaired function. Nat. Immunol..

[49] Leist S.R., Dinnon K.H., Schafer A., Tse L.V., Okuda K., Hou Y.J., West A., Edwards C.E., Sanders W., Fritch E.J. (2020). A Mouse-Adapted SARS-CoV-2 Induces Acute Lung Injury and Mortality in Standard Laboratory Mice. Cell.

[50] Yinda C.K., Port J.R., Bushmaker T., Offei Owusu I., Purushotham J.N., Avanzato V.A., Fischer R.J., Schulz J.E., Holbrook M.G., Hebner M.J. (2021). K18-hACE2 mice develop respiratory disease resembling severe COVID-19. PLoS Pathog..

[51] Dong W., Mead H., Tian L., Park J.-G., Garcia J.I., Jaramillo S., Barr T., Kollath D.S., Coyne V.K., Stone N.E. (2022). The K18-Human ACE2 Transgenic Mouse Model Recapitulates Non-severe and Severe COVID-19 in Response to an Infectious Dose of the SARS-CoV-2 Virus. J. Virol..

[52] Engel K.B., Moore H.M. (2011). Effects of preanalytical variables on the detection of proteins by immunohistochemistry in formalin-fixed, paraffin-embedded tissue. Arch. Pathol. Lab. Med..

[53] Acs B., Pelekanou V., Bai Y., Martinez-Morilla S., Toki M., Leung S.C.Y., Nielsen T.O., Rimm D.L. (2019). Ki67 reproducibility using digital image analysis: An inter-platform and inter-operator study. Lab. Investig..

[54] Baker G.M., Bret-Mounet V.C., Wang T., Veta M., Zheng H., Collins L.C., Eliassen A.H., Tamimi R.M., Heng Y.J. (2022). Immunohistochemistry scoring of breast tumor tissue microarrays: A comparison study across three software applications. J. Pathol. Inform..

