# Supplementary material for: Computational Analysis of Immunohistochemistry Enables Quantitative Assessment of SARS-CoV-2 Nucleocapsid Immunolabeling in Experimentally Infected Mice
Source: Viruses. 2026 Oct 14;18(10):1142. doi: 10.3390/v18101142 (PMC13709754; doi:10.3390/v18101142)
Supplement: Supplementary file 1 [file viruses-18-01142-s001.zip › Supplementary File Legends.pdf]

**Supplementary File S1**. Available results and experimental information details for each mouse included in the study.

**Supplementary File S2**. R session information used for statistical analyses. This file provides the R and RStudio versions, system information, and versions of the packages loaded during the analyses.

**Supplementary Figure S1**. Representative images of the brain histopathological scoring system. Insets represent higher- magnification views of the corresponding areas marked with a yellow square in each corresponding image. Score 0: No lesions; Score 1: Focal presence of a few perivascular lymphocytes; Score 2: Few multifocal areas of inflammation, including a perivascular cuff of up to 3 layers of lymphocytes (arrowhead) and vessels surrounded by fewer lymphocytes (arrow); Score 3: Moderate to abundant multifocal areas of inflammation, including prominent perivascular cuffs (arrowheads). Scale bars in the lower magnification images: 500 μm; scales bars in the insets: 50 μm

**Supplementary Figure S2**. Comparison of immunohistochemical staining with two different antibodies against the SARS-CoV-2 nucleocapsid protein. Lung sections stained with polyclonal (Rockland) and Monoclonal (Cell Signaling) antibodies are shown in images A, C and B, D, respectively. Images C and D correspond to images A and B, respectively, and include digital labelling of areas of positive (red) and negative (blue) immunostaining in the lung parenchyma. Images A and B show similar staining, which is multifocally scattered throughout the parenchyma and predominantly localized within the bronchioles. Images A and B both correspond to a score of 2 in the manual analysis and had SARS-CoV-2 N-positive area values of 1.1% and 1.7% of the lung parenchyma, respectively, in the digital analysis. Scale bars: 1500 μm. Insets of each image correspond to higher-magnification views of the area marked with a yellow rectangle in the corresponding image. Scale bars in the insets: 150 μm. Panels E and F show Spearman's rank correlation analysis between digital quantification of SARS-CoV-2 N-positive area and manual IHC scores in the lung and brain, respectively, with results shown separately for the two antibodies.
